# Adjuvant interferon gamma in patients with drug – resistant pulmonary tuberculosis: a pilot study

**DOI:** 10.1186/1471-2334-4-44

**Published:** 2004-10-22

**Authors:** Roberto Suárez-Méndez, Idrian García-García, Norma Fernández-Olivera, Magalys Valdés-Quintana, María T Milanés-Virelles, Dalia Carbonell, Delfina Machado-Molina, Carmen M Valenzuela-Silva, Pedro A López-Saura

**Affiliations:** 1 "Benéfico Jurídico" Hospital, Havana, Cuba; 2 Center for Biological Research, Clinical Trials Division, Havana, Cuba

## Abstract

**Background:**

Tuberculosis (TB) is increasing in the world and drug-resistant (DR) disease beckons new treatments.

**Methods:**

To evaluate the action of interferon (IFN) gamma as immunoadjuvant to chemotherapy on pulmonary DR-TB patients, a pilot, open label clinical trial was carried out in the Cuban reference ward for the management of this disease. The eight subjects existing in the country at the moment received, as in-patients, 1 × 10^6 ^IU of recombinant human IFN gamma intramuscularly, daily for one month and then three times per week up to 6 months as adjuvant to the indicated chemotherapy, according to their antibiograms and WHO guidelines. Sputum samples collection for direct smear observation and culture as well as routine clinical and thorax radiography assessments were done monthly.

**Results:**

Sputum smears and cultures became negative for acid-fast-bacilli before three months of treatment in all patients. Lesion size was reduced at the end of 6 months treatment; the lesions disappeared in one case. Clinical improvement was also evident; body mass index increased in general. Interferon gamma was well tolerated. Few adverse events were registered, mostly mild; fever and arthralgias prevailed.

**Conclusions:**

These data suggest that IFN gamma is useful and well tolerated as adjunctive therapy in patients with DR-TB. Further controlled clinical trials are encouraged.

## Background

Tuberculosis (TB) is not yet a defeated affection. Although controllable at a community level and curable in individuals, its eradication seems distant. At present, at least one third of the World's population, more than 1 500 million individuals are infected with the *Mycobacterium tuberculosis. *Every year, around 8 – 10 million new cases occur [1]. Two million people die annually due to non-AIDS related TB, which is the highest number of deaths attributable to a single infectious agent [2], and corresponds to the 7^th ^cause of death worldwide [3]. TB represents 26 % of avoidable deaths in developing countries [4]. The emergency of drug resistant (DR) or multidrug-resistant (MDR) strains has increased this global problem, leading to a high morbidity and mortality [5]. MDR-TB affected patients' mean survival ranges from 2 to 14 months [6]. According to the World Health Organization's (WHO) data, MDR patients global proportion is 2.2 % [7].

The infection is mainly transmitted by inhalation of the bacilli coming from respiratory airways infected secretions. Once inhaled, the bacilli are subjected to phagocytosis within the alveolar macrophages, where they can be destroyed. Nevertheless, *Mycobacteria *have developed mechanisms to adapt to the noxious intracellular environment. Thus, it can persist, replicate and disseminate, leading to new infectious foci. Resistance emergence depends on several factors such as initial bacillary load, inadequate or incomplete chemotherapy administration, and the patient's immune condition.

Chemotherapy is successful in most cases, given that the treatment schedule is thoroughly followed. It is prolonged, costly, and needs to be directly observed. Otherwise it is inadequate to kill all the bacilli and drug resistance emerges. Toxicities are frequent as well [8].

The immunologic approach to TB treatment can be promising since only 10 – 20% of infected people develop the disease and many of them have spontaneous remission [9]. Interferon (IFN) gamma plays a main role in the immunity to TB. It is a glycoprotein, secreted by CD4+, CD8+ and NK cells. Nevertheless, CD4+ Th1 lymphocytes are the main producers in response to a stimulus [10].

Enough evidences exist related to IFN gamma action on the macrophages immunoregulatory activity [11-14]]. Lack of production of this cytokine [15] or expression of its receptor [16,17] is associated to the infection's most lethal forms. Interferon gamma has also a potent antifibrotic effect [18-20].

Therefore a pilot clinical study was done with the aim to evaluate IFN gamma effect on drug resistant pulmonary TB patients regarding their clinical, bacteriological and radiological evolutions. The results show that the use of this protein in eight DR-TB patients (four of them MDR) as adjuvant to antibiotics had short and middle-term beneficial effects.

## Methods

An open-label, non-randomized, non-controlled, pilot trial was carried out at the "Benéfico -Jurídico" Hospital, Havana, which is the national reference unit for TB and other respiratory diseases. According to the national TB program, all patients with unfavorable response to treatment are remitted to this center.

The study population was constituted by eight Cuban patients, both sexes, more than eighteen years old, with diagnosis of TB without a favorable response to the usual therapy, who gave their written, informed consent to participate. The diagnosis comprised clinical findings such as cough and expectoration, pulmonary lesions at thorax radiography, and positive DR-TB sputum-smear and culture. To confirm drug resistance the Canetti's multiple proportions method [21] was used as antibiogram. Exclusion criteria were another chronic disease, pregnancy or nursing, severe psychiatric dysfunction, multiple sclerosis or another autoimmune disorder, other pulmonary infections, HIV co-infection, and treatment with glucocorticoids or any other immunosuppressor medication.

The previous drug therapy received by the patients included 4 drugs (isoniazid, rifampin, streptomycin, and pyrazinamide) daily during 2 months, then isoniazid and rifampin twice per week for 2 additional months. Since their sputum tests had not become negative at this point they were returned to the 4 drugs regime plus ethambutol daily for 3 months, and finally isoniazid, rifampin, and ethambutol three times per week for 5 months.

The trial was done in compliance with the Helsinki Declaration. The protocol was approved by the hospital's Ethics Committee and by the Cuban Regulatory Authority. Data from nineteen historical control cases were obtained from the hospital's archives.

Patients received 1 000 000 IU of human recombinant IFN gamma (Heberon Gamma R^®^, Heber Biotec, Havana), intramuscularly, daily during 4 weeks and then 3 times per week for the next 20 weeks. Participants stayed as in-patients during the study period. They received anti-TB drugs (WHO schemes) [22], according to the resistance detected in each case by the antibiogram (Table 1). Drugs were given as follows: rifampin 10 mg/Kg (maximum 600 mg), ethambutol 20 mg/Kg (max. 1500 mg), ethionamide 10 mg/Kg (max. 750 mg), pyrazinamide 15–30 mg/Kg (max. 2000 mg), ciprofloxacin 15–20 mg/Kg (max. 1500 mg), amikacin 15 mg/Kg (max. 1000 mg), and kanamycin 15 mg/Kg (max. 1000 mg) daily. After the end of the 6-months IFN gamma treatment period, chemotherapy continued up to 9 months if the scheme included rifampin and 18 months otherwise.

Evaluations were carried out at entry and monthly during IFN gamma treatment. A complete physical examination was done. Sputum samples were taken for acid-fast-bacilli smear and culture, as well as blood samples for hematological counts, globular sedimentation rate, alanine aminotransferase, and creatinin determinations. Thorax radiographies were also recorded. Afterwards, patients were followed up with half-yearly evaluations during one year.

Treatment efficacy evaluation included clinical, bacteriological and radiological outcomes. Complete response was defined as total disappearance of all signs and symptoms, negative sputum acid-fast-bacilli smear and culture, and pulmonary lesions improvement at X-ray. Partial response included signs and symptoms decrease, negative sputum smear and culture and stable X-ray lesions. No response consisted in signs and symptoms persistence, positive bacteriological examinations, and lesions stabilization or progression. Safety and tolerability of the IFN gamma treatment were monitored by means of a rigorous control of the adverse events that could be presented.

## Results

Eight patients were enrolled in the study. Those were all the pulmonary DR-TB cases in the country during the inclusion period, from December 1999 to February 2002. They had not responded to the usual Directly Observed Treatment Short-course (DOTS) chemotherapy regime. Strain resistance was acquired in all cases. This was well determined since in Cuba all detected cases are screened for resistance on their first isolate. The patients did not have any extra-pulmonary manifestation of the disease. Their demographic and baseline characteristics are shown in Table 1. Five of them were men, six of them non-white. The age ranged between 23 and 54 years old, and body mass index (BMI) between 13.2 and 22.0 Kg/m^2^. Their main symptoms were cough, expectorations, dyspnea, stertors, distal cyanosis, and finger clubbing. Bacteriological tests codification was mostly high and all patients showed active lesions at thorax radiography. Most of them had accelerated globular sedimentation rates (GSR). Anemia or other hematological alterations were not recorded.

**Figure 1:**
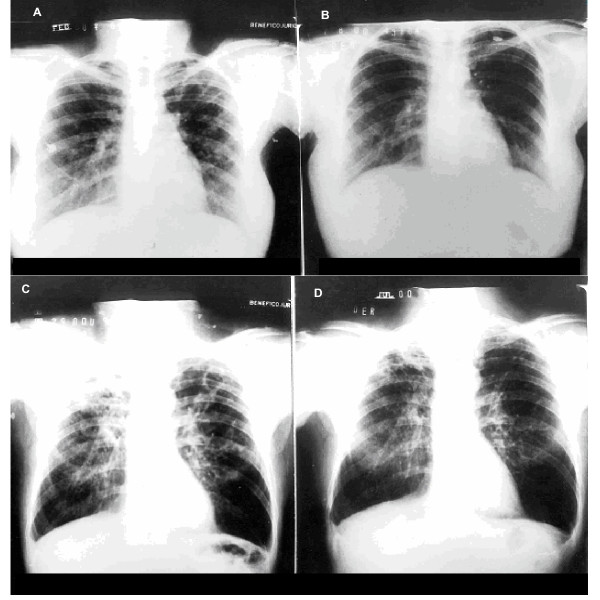
Radiological improvement with IFN gamma treatment (ray-x of two patients are shown). Patient 4 (A), left-lung fibroexudative lesions, and (B) complete resolution after IFN gamma treatment. Patient 2 (C), bilateral moderate exudative lesions before IFN gamma treatment, and (D) important improvement of the lesions afterwards.

A rapid favorable evolution was obtained after treatment with IFN gamma (Table 2). Clinical improvement was evident since the first month of treatment, when all signs and symptoms (except for finger clubbing) had disappeared in all patients and BMI increased in all but one of them. Sputum acid-fast-bacilli smears and cultures were negative since the 1 – 3 months of treatment. The eight patients had radiological improvement, with lesions size reduction (total disappearance in one case) (Figure 1). GSR decreased in five out of 6 patients who had abnormal values at inclusion. After six additional months follow-up, patients # 3 and 4 normalized it to 10 and 15 mm/h, respectively. At the end of the IFN gamma treatment all the patients were evaluated as complete responders.

The treatment with IFN gamma was safe and well tolerated. Four patients presented at least one adverse event. These events were arthralgias, fever, headache and asthenia. All adverse events were mild, except for one moderate fever, which was efficiently controlled with acetaminophen. Significant differences were not detected in other clinical laboratory tests.

After completion or the IFN gamma 6-months treatment the patients continued with the corresponding chemotherapy schedule. Seven of the eight patients remained bacteriologically, clinically and radiologically negative at least twelve months after the treatment with IFN gamma concluded. However, patient number five relapsed six months after the end of IFN therapy. He developed additional resistance to rifampin and ethionamide and a chronic obstructive respiratory disease that contributed negatively to his evolution.

Table 3 shows the results obtained at the same hospital with the 19 DR-TB cases during the five years prior to the present study. These patients had also failed to the standard DOTS regime. They were all resistant to isoniazid and rifampin, 13 were resistant to streptomycin, 7 to kanamycin, 4 to ethambutol, and one to pyrazinamide. Resistance was primary in 2 cases and acquired in the rest. Their average age was 58 years. Management was essentially the same as for the patients included in the study, except for IFN gamma treatment. None of these DR-TB cases reached culture conversion at three months of treatment with chemotherapy and less than half had converted at six months. Their clinical outcome was also worse.

## Discussion

In spite of the reduced size of the population studied, the results suggest the efficacy of IFN gamma on DR-TB, when used as adjuvant to chemotherapy. All eight patients were considered as complete responders at the end of IFN treatment with disappearance of the disease signs and symptoms, sputum tests conversion, and pulmonary lesions improvement. Bacteriological and radiological improvement correlated with the clinical evolution; BMI increased and manifestations as cough and expectoration did not recur after IFN gamma treatment. Clinical practice demonstrates that these results are very difficult to obtain in such a short period of time with the chemotherapy alone.

Any conclusion from this study is also limited by the fact that it was not a controlled trial. This was not possible due to the low incidence of TB (7.6/100,000 inhabitants in 2002) [23] and DR-TB in Cuba [7]. Therefore a historical control with the results obtained at the same hospital by the same investigators in patients treated only with chemotherapy is used for comparison. Despite the fact that this kind of control is not fully comparable since they were not in the original design of the trial, a clear difference is shown regarding patients' performance. Literature reports on chemotherapy-treated DR-TB patients show similar unfavorable outcome [6].

The fact that four patients received rifampin, according to the strain sensitivity, which is one of the election drugs for the treatment of TB with excellent results in patients treated with DOTS regime, does not diminish the consideration regarding the possible benefit exerted by IFN gamma since these same patients had already not responded to this antibiotic as part of the DOTS. Moreover, one patient recurred after he had finished IFN gamma treatment for 6 months, despite still being under chemotherapy regime. This suggests that in his case a new IFN gamma cycle combined to the antibiotics would have been necessary, but this was not previewed in the protocol. However, only a controlled trial can definitely clarify the role of IFN gamma and second line antibiotics in this kind of patient's improvement.

The radiological results demonstrated IFN gamma antifibrotic properties as well. All patients had a reduction in the pulmonary lesions size, while one showed a complete resolution. This effect cannot be attributable to the antibiotics, since it is well known that DR-TB patients only develop radiological improvement long time after sputum smears and culture become negative. In many cases extensive fibrotic lesions never improve, and stay stable for life. This antifibrotic action agrees with that obtained with IFN gamma in idiopathic lung fibrosis [24] and suggests that IFN gamma can have future indications in other pulmonary diseases where fibrosis is present.

Therapy was well tolerated. It was not necessary to suspend the combined treatment due to adverse events; mostly mild. Adverse events such as arthralgias, fever and headache coincide with those reported for interferons [25].

Immunity to TB depends on the development of CD4+ cells- and macrophages-mediated Th1 response. The role of IFN gamma as the main macrophage-activator Th1 cytokine has been clearly established in animal models infected with *M. tuberculosis *[15,26,27]. IFN gamma action on the macrophages leads to kill intracellular *Mycobacteria*. It stimulates macrophages to produce tumor necrosis factor alpha (TNF α), oxygen free radicals and nitric oxide, increase surface display of MHC antigens and Fc receptors, decrease lysosomal pH, and increase the intracellular concentration of some antibiotics [11-14,28].

Regarding its antifibrotic effect, IFN gamma inhibits lung fibroblast proliferation and chemotaxis in a dose dependent manner, and reduces collagen synthesis [18,19,29]. Furthermore, this protein is a potent inhibitor of the transforming growth factor β (TGF-β)[20], involved in the pathogenesis of many fibrotic lung diseases [30-32].

On the other hand, mutations in the IFN gamma gene [15], or in the IFN gamma receptor alpha chain gene [16,17], increase susceptibility to develop the disease. Patients with disseminated BCG and other infections present defects in IFN gamma and other cytokines secretion and action [33-35].

IFN gamma therapy has been shown effective for cerebral tuberculosis caused by a multidrug-resistant strain [36]. The aerosol route of administration has been proposed as organ specific delivery method, obtaining a high release to infected alveoli [37]. Condos et al. reported clinical and bacteriological improvement and tolerability with aerosolized IFN gamma in five patients with MDR-TB [38]. In addition, other previous trials demonstrated promising results in patients infected with other *Mycobacteria *[10,39-42].

## Conclusions

These results can suggest a beneficial effect of IFN gamma when it is used as adjuvant in the treatment of tuberculosis patients that have resistance to standard chemotherapy, and encourage carrying out more extensive, controlled studies. Combination with second-line drugs can reduce the time of treatment, diminishing toxicities and possible relapses; in many cases could reduce the application of recessional surgery. Further controlled clinical trials are needed to confirm these results.

## Competing interests

Authors IGG, CMVS, and PALS are employees of the Center for Biological Research, which is part of the Center for Genetic Engineering and Biotechnology, Havana network, where IFN gamma is produced. The rest of the authors have no competing interests at all.

## Authors' contributions

RSM conceived the study and carried out the bacteriological determinations. IGG participated in the study design and coordinating, and wrote the manuscript draft. NFO, MVQ, MTM, DC, and DMM took care of patient recruitment, management, and follow-up. CMVS participated in the study design and result analysis. PALS took part in the design, results analysis and manuscript writing. All authors read and approved the final manuscript.

## Pre-publication history

The pre-publication history for this paper can be accessed here:



## Figures and Tables

**Table 1:** Demographic and baseline characteristics of patients with DR-TB treated with IFN gamma

**Patient**	**1**	**2**	**3**	**4**	**5**	**6**	**7**	**8**
**Age (years)**	28	50	54	27	44	50	23	37
**Sex**	Male	Male	Female	Female	Male	Male	Female	Male
**Race**	Mestizo	White	Mestizo	Mestizo	White	Mestizo	Black	Black
**BMI (Kg/m**^2^**)**	19.6	18.9	13.2	18.3	14.6	21.6	22.0	17.1
Sputum smear status*	0	9	9	9	8	9	8	9
Sputum culture status**	7	9	7	9	6	9	8	9
**Drug Resistance**	INH RIF STR	INH STR	INH RIF STR	INH RIF	INH, STR	INH RIF STR	INH	INH
**Initial thorax X-ray**	R1	R2	R3	R4	R5	R6	R7	R8
**GSR (mm/h)**	5	66	53	53	68	101	10	60

INH: Isoniazid; RIF: Rifampin; STR: Streptomycin; ETB: Ethambutol; ETN: Ethionamide; PRZ: Pyrazinamide; CPF: Ciprofloxacin; KAN: Kanamycin; AMK: Amikacin.

* Number of bacilli; 0: 0 in 4 lines; 8: 25 or more in 1 line; 9: bacilli in most of the fields.

** Number of colonies; 6: 6–24 colonies; 7: 25–100 colonies; 9: confluent growth.

R1 and R3. Extensive bilateral exudative lesions

R2: Moderate exudative lesions

R4: Left fibro-exudative lesions

R5: Moderate Bilateral fibro-exudative lesions.

R6: Cavitary lesions at superior lobes; fibrous lesions at right superior lobe; alveolar infiltrate at left superior lobe.

R7 and R8: Very diffuse fibromatous lesions at superior lobes.

**Table 2:** Six months follow-up data of DR-TB patients treated with IFN gamma

**Patient**	**1**	**2**	**3**	**4**	**5**	**6**	**7**	**8**
**Drug regimen**	ETB		ETB	ETB		ETB		
		RIF			RIF		RIF	RIF
	ETN		ETN	ETN		ETN		
		ETB			ETB		ETB	ETB
	PRZ		PRZ	PRZ		PRZ		
		PRZ			PRZ		PRZ	PRZ
	CPF		CPF	CPF		CPF		
		KAN			KAN		KAN	KAN
	KAN		KAN	AMK		KAN		
**Gain BMI (Kg/m^2^)**	1.8	0.4	0.4	0.4	0.3	2.2	1.8	- 2.1^!^
**Sputum smear status**	Negative	Negative	Negative	Negative	Negative	Negative	Negative	Negative
**Sputum culture status**	Negative	Negative	Negative	Negative	Negative	Negative	Negative	Negative
**Conversion time**	2 mo.	3 mo.	3 mo.	1 mo.	3 mo.	2 mo.	2 mo.	3 mo.
**Thorax X-ray**	Residual fibrosis	Reabsorption and residual fibrosis	Residual bilateral fibrosis.	Lesions resolution	Residual fibrosis	Lesions size reduction	Residual fibrosis	Lesions size reduction
**GSR (mm/h)**	5	48	26	40	42	20	23	77

Legend: see Table 1

! Lost body weight

**Table 3:** Bacteriological evolution of DR-TB historical controls at "Benéfico Jurídico" Hospital (1994–1999) under specific chemotherapy. These patients had failed to respond to the standard DOTS regime and were then further treated with chemotherapy according to the sensitivity of their strains.

	**Sputum acid-fast-bacillus smear status**				**Sputum culture status**			
	
	**Positive**		**Negative**		**Positive**		**Negative**	
**Trimester**	**N**	**%**	**N**	**%**	**N**	**%**	**N**	**%**

**1**	17	89.5	2	10.5	19	100	-	-
**2**	5	26.3	10	52.6	8	42.1	7	36.8
**3**	2	10.5	9	47.4	2	10.5	9	47.4
**4**	2	10.5	9	47.4	2	10.5	9	47.4

Five patients died and other three interrupted the chemotherapy before concluding it. Percents are calculated considering initial DR-TB population (19 patients)
